# Managers’ attitudes to depression and the association with their rating of how work capacity is affected in employees with common mental disorders

**DOI:** 10.1186/s13104-024-06750-7

**Published:** 2024-05-21

**Authors:** Jenny Hultqvist, Gunnel Hensing, Lisa Björk, Monica Bertilsson

**Affiliations:** 1 Department of Health and Rehabilitation, Institute of Neuroscience and Physiology, The Sahlgrenska Academy, University of Gothenburg, 405 30 Gothenburg, Sweden; 2 School of Public Health and Community Medicine, Institute of Medicine, The Sahlgrenska Academy, University of Gothenburg, 405 30 Gothenburg, Sweden; 3 Region Västra Götaland, The Institute of Stress Medicine, 413 19 Gothenburg, Sweden; 4 Department of Sociology and Work Science, University of Gothenburg, 405 30 Gothenburg, Sweden

**Keywords:** Attitudes, Common mental disorder, Employee, Manager, Work capacity

## Abstract

**Objective:**

This explorative, cross-sectional study assessed the association between managers’ attitudes to employee depression and their rating of how common mental disorders (CMDs) affect employee work capacity.

**Results:**

A principal component analysis was performed for the nine variables concerning managers’ rating of how CMDs can affect work capacity among employees. The analysis resulted in two factors: task-oriented- and relational work capacity. The result of the multivariate analysis of covariance showed a *p* value of 0.014 (Pillai’s trace) indicating a statistically significant association between managers’ attitudes towards employee depression and managers’ rating of how CMDs affect work capacity. The association was significant for both factors as indicated by the *p* value of 0.024 for task-oriented work capacity and the *p* value of 0.007 for relational work capacity. The R^2^ value was 0.022 for task-oriented work capacity and 0.017 for relational work capacity. We assumed that negative attitudes towards employee depression would be associated with a perception of decreased work capacity among employees with CMDs. The results showed a significant association; however, the effect (~ 2%) was small. Further studies of manager’s attitudes and other possible determinants of managers’ rating of CMD-related work capacity are needed to better understand these factors.

**Supplementary Information:**

The online version contains supplementary material available at 10.1186/s13104-024-06750-7.

## Background

Managers have a key role in occupational health with responsibilities for safety, prevention, and rehabilitation [1]. However, research on managers’ attitudes towards common mental disorders (CMDs) is scarce even though these disorders are one of the most prevalent occupational health problems in western countries [2, 3]. In a previous focus group study, we explored managers’ (*n* = 31) experience-based understanding of how CMDs affect employees’ capacity to work [4]. Five categories were identified: cognitive capacities, time management, work independence, flexibility, and social interactions. According to the managers, these changes in capacity to work among employees had negative consequences for work performance and output, and workplace and manager–employee interaction [4]; these findings are reflected in other qualitative studies [5, 6]. The qualitative findings of are to to a large extent in line with the results of a quantitative study [7] showing that multiple dimensions of work performance were impaired by CMD; mental-interpersonal tasks, time management, output (e.g. handling the workload and finishing work on time), and physical tasks.

Given the high prevalence of CMDs in the working-age population and the possible negative consequences for work performance, work participation and productivity, CMDs and work capacity are highly relevant issues for employers and for society at large.

In previous quantitative studies, we have shown that male Swedish managers were more likely to have more negative attitudes towards depression than their female counterparts, even after controlling for several other factors that might confound the association [8]. Also there was an association between managers’ negative attitudes to depression and managers’ reporting that they found out about employees’ CMD from self-disclosure from the employee to a lesser extent [9]. We have also found that managers with negative attitudes to employees with CMD were less likely to have taken managerial preventive actions, specifically reviewing assignments and work situation, and talking about CMD at the workplace [10]. Other studies have reported that managers’ negative attitudes towards CMD can affect managers’ behaviour and actions regarding supportive practices and hiring decisions [11, 12].

A Finnish study with a general population sample (*n* = 10,000) aged 15–80 years showed that 35–58% perceived people with depression as weak and 58% thought that people with depression should “pull themselves together” [13]. It can be assumed that such societal attitudes may influence managers’ perceptions of work performance and work capacity in employees with CMDs.

The present study draws upon previous research [4–6, 8–12], assuming that negative attitudes towards employee depression would be associated with a perception of decreased work capacity in employees with CMDs. Research on stigmatizing attitudes towards mental illness often focuses on individual factors rather than contextual factors [13–14]. According to Johns [15], the workplace context can affect the occurrence of attitudes and behaviours in organizations.

Thus, the aim of this explorative study was to assess the association between attitudes to depression and managers’ rating of how CMDs affect work capacity while also taking contextual factors into consideration.

The following research question was addressed:

To what degree are managers’ attitudes to employee depression associated with managers’ rating of how CMDs affect work capacity?

## Methods

An explorative, cross-sectional study was considered relevant. This is a part of the New Ways research programme on mental health at work and the sub-project “Managers’ perspective– a missing piece” [8–10, 16]. In 2017, Swedish managers were invited to take part in a web-based survey.

An explorative, cross-sectional study was considered relevant. This is a part of the New Ways research programme on mental health at work and the sub-project “Managers’ perspective– a missing piece” [8–10, 16]. In 2017, Swedish managers were invited to take part in a web-based survey.

### Study sample

**Fig. 1:**
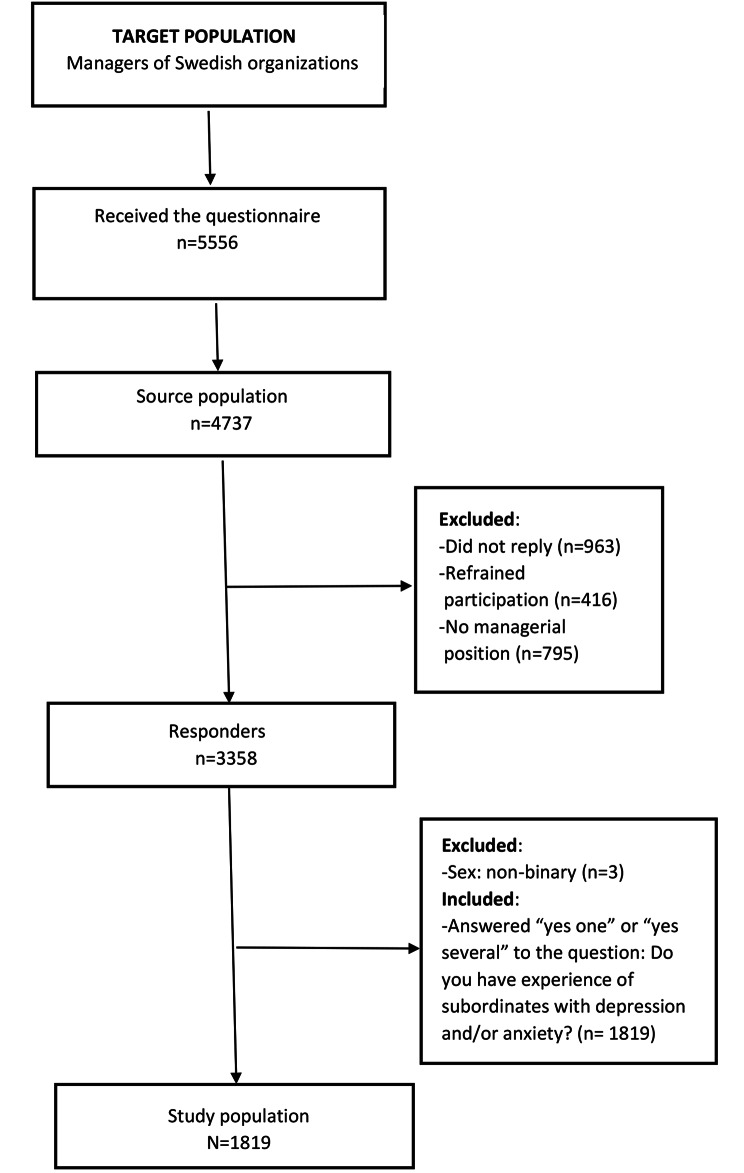
Flowchart for selection of the study population of Swedish managers. Data was collected in 2017

Participants were recruited through The Citizen Panel, Laboratory of Opinion Research (LORE) at the University of Gothenburg, Sweden (*n* = 5000) (https://www.gu.se/en/som-institute/the-swedish-citizen-panel/citizen-panel-for-researchers) and the HELIX Competence Centre at Linköping University, Sweden (*n* = 556) (https://liu.se/en/research/helix-competence-centre). Participation was based on written informed consent. The initial question “I am not a manager” resulted in the exclusion of 795 individuals. Due to invalid e-mail addresses or technical errors, another 24 individuals were excluded, leaving 4737 eligible participants. Of these, 3358 participated and constituted the study population. An inclusion criterion for the present study was having experience of CMDs among employees at their current workplace in the last 2 years. The final study sample consisted of 1819 participants (Fig. 1); 927 respondents (51%) had experience of one employee with a CMD, and 892 respondents (49%) had experience of two or more employees with a CMD. The study sample included senior managers (such as administration manager, managing director), middle management (manager of managers), first-line managers, group leaders/supervisors and expert/operations managers (such as personnel manager, finance manager). Descriptive information on the participants is presented in Table 1.

**Table 1:** Descriptive statistics of the study sample of Swedish managers (N = 1819). The data was collected in 2017

Variable	n(%)	Missing
Women/Men	694(38)/1117(62)	8
*Age*: 20–29 years 30–39 years 40–49 years 50–59 years 60 years and older	29(2) 266(15) 590(32) 676(37) 257(14)	1
*Level of education*: Upper secondary school or lower Degree from college/university Other post-secondary education	247(14) 1172(64) 398(22)	2
*Organizational size (i.e. number of staff members)*: 0 to 9 10 to 49 50 to 250 251 to 1000 more than 1000	175(10) 335(18) 359(20) 281(15) 668(37)	1
*Number of employees per manager*: 0 1-5 6-10 11-20 21-30 31-40 41-50 more than 50	135(7) 485(27) 408(23) 366(20) 147(8) 108(6) 65(4) 98(5)	7

### Dependent variable

The dependent variable was the association between attitudes to depression and managers’ rating of how CMDs affect work capacity. This was measured using nine variables specifically designed for the survey. The variables were derived from an validated instrument, the Capacity to Work Index (C2WI) described elsewhere [17]. The C2WI was developed from qualitative studies of individuals with their own experience of working with CMDs [18–20].

The nine variables consisted of statements with the following response options: 1, not affected at all; 2, became somewhat more difficult; 3, became quite difficult/much more difficult; and 4, do not know. The “do not know” option offered valuable descriptive information and was included in the survey to increase validity; however, this option was excluded in the inferential analysis in the present study.

A principal component analysis with varimax rotation with Kaiser normalization was performed for the nine variables concerning managers’ rating of how CMDs can affect work capacity among employees which was the targeted outcome. The analysis resulted in components loading on two factors: task-oriented work capacity and relational work capacity.

### Independent variable

Negative attitudes were measured using the Managerial Stigma towards Employee Depression (MSED) instrument [12, 21]. The MSED addresses stigma and its potential stereotypes, prejudices and discrimination by measuring affective, cognitive and behavioural attitudes towards employees with depression. The MSED comprises 12 items with statements reflecting managers attitudes on a 6-point Likert scale from 1 (strongly disagree) to 6 (strongly agree). A Swedish version of the MSED instrument was developed for the main project [8]. The Swedish version has been tested for internal consistency and found to be sufficient (α = 0.80) [8].

### Covariates

Based on an earlier study, we identified four covariates [b]: sex, level of education, industry, and size of company. The response options for sex were “women”, “men”, and “non-binary”. These were dichotomized into “men” and “women,”. Only three respondents indicated non-binary and were therefore excluded. Level of education included five response options: “compulsory school”, “upper secondary school or equivalent”, “degree from college/university (minimum 3 years)”, and “other post-secondary education”. This was dichotomized into “secondary school or lower” and “post-secondary school” for the analysis.

Industry was assessed with the question “in which industry does the company’s/organization’s main activity belong?”. In accordance with the Swedish Standard Industrial Classification (https://www.scb.se/en/documentation/classifications-and-standards/swedish-standard-industrial-classifcation-sni/), 16 different industries were clustered into three categories according to the people-data-things hierarchy [22]. “Blue collar” refers to industries working with things, “white collar” refers to industries working with data, and “pink collar” refers to industries working with people. A fourth category “other type” was used for those industries not fitting into one of the three categories. In this study, the category “other type” was not included. A question on the total number of employees in the organization was used to represent the size of the company. The response options “0–9”, “10–49”, “50–250” and “251–1000” and “more than 1000” were grouped into “≤250” and “≥251”, respectively.

The questions from the survey that were used in this study are presented in the Supplementary file.

### Statistical analysis

First, the correlation between managers’ attitudes to employees with depression and the two factors representing the association between attitudes to depression and managers’ rating of how CMDs affect work capacity was explored using Spearman’s rho. A non-parametric test was chosen because the data on attitudes was not normally distributed.

Second, a multivariate analysis of covariance (MANCOVA) was performed to further investigate how managers’ attitude towards employee depression was associated with managers’ attitudes to depression and managers’ rating of how CMD affect work capacity. Four co-variates were included simultaneously in the analysis: sex, level of education, industry, and size of company. In the analysis, the summed variable managers’ attitudes towards employees with CMD (i.e. the independent variable) was recoded into four groups based on the quartiles.

An expert statistician was consulted regarding data management and statistical methods. All analyses were done using IBM SPSS Statistics version 27. A p value < 0.05 was considered statistically significant.

## Results

The results showed a significant, but weak, correlation (*p* = 0.012, *r*_s =_ 0.060) between managers’ attitudes to depression among employees and relational work capacity. Regarding task-oriented work capacity, the association was non-significant (*p* = 0.054, *r*_s =_ 0.046). However, there was a strong correlation between task-oriented work capacity and relational work capacity (*p* < 0.001, *r*_s_ =0.510); accordingly, the choice was to include both factors in a subsequent analysis [23].

The results of the MANCOVA showed a *p* value of 0.014 (Pillai’s trace), indicating a statistically significant association between managers’ attitudes towards employee depression and managers’ rating of how CMDs affect work capacity. The association was significant for both factors as indicated by the *p* value of 0.024 for task-oriented work capacity and the *p* value of 0.007 for relational work capacity. R^2^ for task-oriented work capacity was 0.022 and 0.017 for relational work capacity. Accordingly, managers’ attitudes explained approximately 2% of variance in the dependent variable.

## Discussion

We assumed that negative attitudes towards employee depression would be associated with a perception of decreased work capacity in employees with CMDs. The results showed a significant association; however, managers´attitudes only explained ∼ 2% of the variance in managers´rating of work capacity in employees with CMDs. This result warrants some consideration.

Research on stigmatizing attitudes towards mental illness often focuses on individual factors rather than contextual factors [14]. According to Johns [15], there are two levels of organizational context: a broadly conceptualized context and more particular contextual variables that shape behaviour or attitudes. Johns [15] argues that more particular contextual variables, for example, social structure and social influence, are ingrained in the broader context. Therefore, the workplace context can affect the occurrence of attitudes and behaviours in organizations. Two of the broadly described context variables according to Johns [15] are “occupation” and “place”. In this study, we controlled for industry (related to occupation) and the size of company (related to place). We also controlled for personal factors, sex and level of education, which have both shown an association with managerial stigma towards employees with depression [7, 11]. Controlling for these contextual and personal covariates should strengthen the association between managers’ attitudes towards employee depression and their rating of work capacity among employees with CMDs. Even so, the total effect i.e. was marginal at ∼2%. In another explorative study controlling for various covariates, we found no association between managers’ attitudes to employee depression and recommendation of sick leave in response to a CMD-labelled video case vignette [16]. Both results contrast with research on stigmatizing attitudes in a work context and the association with adverse work outcomes for persons with mental illness or mental health issues [10]. However, that research [10] included people with CMDs and severe mental disorders. Putting these two groups together overlooks the fact that symptom severity and the type of diagnosis may be associated with the degree of stigmatizing attitudes [24].

As measured in the present study, the importance of managers’ attitudes to employee depression is downplayed, which, in relation to previous research on managers’ attitudes to employee depression is a positive finding [10–12]. Our study seems to be the first exploring the association between managers’ attitudes to depression and managers’ rating of how CMDs affect employee work capacity. The results should be interpreted with caution given the low effect size. Further studies of managers’ attitudes and other possible determinants of managers’ rating of CMD-related work capacity are needed to better understand these factors. Increased understanding of such factors could influence managerial training and support more positive and health-promoting work environments for employees with CMD.

A strength of the study was the use of a validated instrument to measure managers’ attitudes towards employees with depression [21]. In addition, the questions regarding work capacity in relation to CMD were derived from a validated instrument [17]. Further strengths of the study were the large sample size of managers with experience of employees with CMDs and the inclusion of managers from a variety of work sectors and industries.

### Limitations

The study’s cross-sectional design prevents causal inferences, and the study sample was not selected randomly. In future studies a randomized sample of managers should be used. The sample is biased towards more well-educated managers. This implies a probable under-representation of participants with negative attitudes. However, this study included a range of attitudes from negative to positive.

### Electronic supplementary material

Below is the link to the electronic supplementary material.

Supplementary Material 1

## Data Availability

The data used for this study have been archived at the Laboratory of Opinion Research (LORE) at the University of Gothenburg and can be obtained by contacting LORE at info@lore.gu.se.

## Abbreviations

CMD: common mental disorders
MANCOVA: multivariate analysis of covariance
MSED: managerial stigma towards employee depression
