# Supplementary material for: Managers’ attitudes to depression and the association with their rating of how work capacity is affected in employees with common mental disorders
Source: BMC Res Notes. 2024 May 21;17:144. doi: 10.1186/s13104-024-06750-7 (PMC11110306; doi:10.1186/s13104-024-06750-7)
Supplement: Supplementary file 1 — Supplementary Material 1 [file 13104_2024_6750_MOESM1_ESM.docx]

# Questions from the eu35_manager online survey

q10 What is your gender?

o Woman (1)

o Man (2)

o Non-binary (3)

q12 What is the highest level of education you have completed? Select the answer that you think best applies to you.

o Compulsory school (1)

o Upper secondary school or equivalent (2)

o Degree from college/university (minimum three years) (3)

o Other post-secondary education (4)

q18 In which industry does the company’s/organization’s main activity belong? Select the answer that you think best applies to you.

o Agriculture, forestry, fishing (1)

o Mineral extraction (industry) (2)

o Manufacturing industry (3)

o Construction and craftsmanship (4)

o Provision of electricity, heat, water, sewage, waste (5)

o Trade/commerce (6)

o Transport (7)

o Hotel and restaurant operations (8)

o IT, information, and communications activities (9)

o Financial and insurance activities (10)

o Education (11)

o Health care, social services (12)

o Public administration and defense (13)

o Legal, economic, scientific, and technological activities (14)

o Culture, entertainment, recreation (15)

o Other type of activity (16)

q20 In total, how many staff members are there in your company/organization?

o 0 – 9 (1)

o 10 – 49 (2)

o 50 – 250 (3)

o 251 – 1,000 (4)

o More than 1,000 (5)

q52 During the past two years, have you had staff members at your current workplace who have had depression and/or anxiety disorders?

The question also applies to those who have worked less than two years at the current workplace.

o Yes, several staff members (1)

o Yes, one staff member (2)

o No, no staff member (3)

o Don't know (4)

q33 Depression today is a common occurrence at work. The following questions are about your general perception of depression at work. To what extent do you agree with the following statements?

The questions on attitudes towards employee depression belong to the “Managerial stigma towards employee depression” instrument measuring managers’ affective, cognitive and behavioral attitudes to employees with depression. References:

Martin A. Individual and contextual correlates of managers' attitudes toward depressed employees. Hum Resour Manage. 2010;49(4):647–668.

Martin AJ, Giallo R. Confirmatory factor analysis of a questionnaire measure of managerial stigma towards employee depression. Stress Health. 2016;32(5):621–628

Mangerini I, Bertilsson M, de Rijk A, Hensing G. Gender differences in managers’ attitudes towards employees with depression: a cross-sectional study in Sweden. BMC Public Health. 2020;20(1):1–15.
